# Review of cancer treatment with immune checkpoint inhibitors: Current concepts, expectations, limitations and pitfalls

**DOI:** 10.1007/s00508-017-1285-9

**Published:** 2017-11-02

**Authors:** Christiane Thallinger, Thorsten Füreder, Matthias Preusser, Gerwin Heller, Leonhard Müllauer, Christoph Höller, Helmut Prosch, Natalija Frank, Rafal Swierzewski, Walter Berger, Ulrich Jäger, Christoph Zielinski

**Affiliations:** 1 Clinical Division of Oncology, Department of Medicine I, General Hospital, Währinger Gürtel 18–20, 1090 Vienna, Austria; 2 Central European Cooperative Oncology Group, Vienna, Austria; 3 Comprehensive Cancer Center, General Hospital, Medical University Vienna, Vienna, Austria; 4 Department of Pathology, General Hospital, Medical University Vienna, Vienna, Austria; 5 Department of Dermatology, General Hospital, Medical University Vienna, Vienna, Austria; 6 Department of Radiology, General Hospital, Medical University Vienna, Vienna, Austria; 7 European Cancer Patient Coalition (ECPC), Brussels, Belgium; 8 Institute for Cancer Research, Department of Medicine I, Medical University Vienna, Vienna, Austria; 9 Clinical Division of Hematology, Department of Medicine I, Medical University Vienna, Vienna, Austria

**Keywords:** Cancer, Immunotherapy, PD-1, Experts, Review

## Abstract

Immunotherapy by checkpoint inhibition is about to profoundly change cancer therapy. The number of indications are growing at an unprecedented speed. Clinical studies have demonstrated efficacy in a variety of solid tumors and in hematologic malignancies, although some clinical trials have produced negative results. Thus, it is fair to assume that there are obvious limitations and pitfalls in immunotherapy. Future concepts for combination treatment of immune checkpoint inhibitors have to be developed, but there is also urgent need for better and standardized biomarkers to identify those cancer patients who will benefit from treatment by checkpoint inhibition. The current overview summarizes current knowledge on immune checkpoint inhibitor treatment in malignancies, its outlook and limitations, diagnostic means and, finally, side effect management.

## Introduction

In the treatment of solid malignancies, immune checkpoint inhibitors constitute an important breakthrough positively influencing treatment outcomes regarding progression-free (PFS) and/or overall survival (OS), as compared to chemotherapy-based treatment. Immune checkpoint inhibitor treatment involves antibodies generated against the cytotoxic T‑lymphocyte associated protein 4 (CTLA-4), the programmed death receptor 1 (PD-1) or its ligand (PD-L1). Thus, immune checkpoint inhibitors modulate the interaction between tumor cells and cytotoxic T lymphocytes in the tumor environment, which are exhausted in their function. Targeting with CTLA-4 or PD-1 or PD-L1 reverses the exhaustion of cytotoxic T lymphocytes thus leading to the elimination of tumor cells via the re-induction of the “natural” function of the T cell population. Interestingly, some of the clinical results when using anti PD-1 and anti PD-L1 antibodies may be also due to additional effects on T‑cells including their targeting of B7.1 [1–4].

Whereas anti-CTLA-4 antibodies (ipilimumab and tremelimumab), anti-PD-1 antibodies (nivolumab and pembrolizumab), and anti-PD-L1 antibodies (atezolizumab, avelumab and durvalumab) have produced remarkable results regarding tumor control in many malignancies (albeit in various treatment lines), response is often followed by relapse and disease progression. Conversely, cancer patients who benefit from checkpoint inhibition can achieve long-term benefit and remarkable duration of PFS. Inhibition of CTLA-4 by ipilimumab represents the first compound ever used in immune checkpoint inhibitor treatment to enter clinical routine. Ipilimumab was licensed for use in advanced metastatic melanoma [5]. The second group of drugs for immune checkpoint inhibition were anti PD-1 or anti PD-L1 antibodies which are currently registered by the U.S. Food and Drug Administration (FDA) for metastatic malignant melanoma, non-small cell lung cancer (NSCLC), renal cell cancer (RCC), head and neck cancer (HNSC), urothelial carcinoma and Hodgkin’s lymphoma in various stages of the respective disease and in the context of varying treatment histories [6–12]. Many other malignancies (e. g. hepatocellular carcinoma, ovarian cancer, mesothelioma, gastric cancer, B‑cell non-Hodgkin lymphoma) are currently under clinical investigation to determine a possible efficacy of checkpoint inhibition [13–15]. Landmark trials have shown significant improvement of survival rates in many advanced metastatic cancers (e.g. NSCLC, HNSC, metastatic malignant melanoma and renal cell carcinoma), when compared with chemotherapy or, as in RCC,other treatment modalities, such as everolimus in second line.

## Current indications for immune checkpoint inhibitor treatment

Indications for immune checkpoint inhibitor treatment are rapidly increasing [16]: while PD-1 and CTLA-4 directed antibodies were primarily registered for the treatment of metastatic malignant melanoma, NSCLC followed quickly with currently nivolumab, pembrolizumab and atezolizumab licensed for second line treatment, and pembrolizumab for first line treatment for patients with NSCLC and PD-L1 expression of >50%. Additional indications currently comprise renal and urothelial cancer, HNSC squamous epithelial cancer and Hodgkin’s lymphoma.

## Hematologic disorders

In hematology, the checkpoint inhibitors nivolumab and pembrolizumab have been approved for the treatment of Hodgkin’s disease [17]: Classical Hodgkin’s lymphoma cells show high PD-L1 expression. Clinical studies have been primarily performed in patient groups with relapsed disease after autologous stem cell transplantation ineligible for autologous stem cell transplantation, a relapse after brentuximab vedotin therapy or without this treatment. The outcome was remarkable [18, 19]: overall response rates (ORR) were up to 65% and complete remission (CR) occurred in approximately 20% of patients. Preliminary results from a phase 1 and 2 study of brentuximab vedotin in combination with nivolumab in patients with relapsed or refractory Hodgkin’s lymphoma showed ORR of 90% (26/29) and CR of 62% (18/29).

Many clinical studies with checkpoint inhibitors are currently under way to test their efficacy in various other hematologic indications either as monotherapy or, as in the case of multiple myeloma, in combination with immunomodulators, such as lenalidomide and pomalidomide [20].

Further concepts applied in the field of hematologic malignancies look at the combination of immune checkpoint inhibitors with other immunotherapies: primarily, combinations with bispecific T‑cell engaging antibody (BITE/blinatumomab) or with chimeric antigen receptor T cells (CAR-T-cells) [21]. Although both treatments depend on functional T‑cells, immune checkpoint inhibitors could enhance the T‑cell response to make BITE or CAR-T-cell therapy more effective [11, 22, 23]. In childhood relapsed or refractory acute lymphocytic leukemia, the combination of CAR-T-cell immunotherapy and checkpoint inhibition showed an overall remission rate of 82% within 3 months and an OS of 89% at 6 months.

## Predictive biomarkers

For anti CTLA-4 therapy, some biomarkers have been identified which may be useful for predictive purposes, but none has entered clinical routine or should be used before starting treatment.

### PD-L1

For anti-PD1 and anti-PD-L1 therapies, mainly immune cell-associated PD-L1 expression has been identified to represent an important predictive enrichment biomarker. Although widely accepted, many important questions remain open regarding the generalization of PD-L1 being a reliable biomarker: thus, PD-L1 expression is induced by cytokines therefore generating only a “snapshot” of the overall biological situation [24]. As a further limitation, various cut-off values of PD-L1 expression have been used in various clinical trials varying between 1, 5, 10, 25 and 50%. Considering the current state of knowledge it seems acceptable to generalize, however, that higher PD-L1 values might indicate a better response to therapy leading to the fact that PD-L1 is currently accepted as the best available enrichment biomarker [25, 26]. In addition, other immune-related biomarkers are also currently being studied and may be of predictive importance, which mainly include intratumoral CD8+ T‑cell infiltration as a manifestation of the immunogenicity of a certain tumor [27].

### Mutational load and molecular alterations

Another group of predictive biomarkers could be derived from the assessment of the “mutational landscape” (mutational load) of a tumor: this assumption was first corroborated by an early publication on the efficacy of nivolumab in NSCLC, and more recently in a study with pembrolizumab in NSCLC with more than 200 mutations in protein-encoding regions of the tumor genome [28]; however, this field is evolving relatively slowly, and there is no established cut-off value, which would be clinically applicable [29, 30].

In a related context and regarding mismatch repair-deficient cancers, microsatellite instability (MSI-H) could predict response to checkpoint inhibition therapy in colorectal cancer [31]. Some 15% of colorectal cancers and some 22% of endometrial and gastric carcinomas show MSI-H frequencies of >10%. These tests might, therefore, be of value in treatment decisions for the use of immune checkpoint inhibitor treatment in cancers of the gastrointestinal tract [32]. At present, PD-L1 immunohistochemistry and MSI status assessed in all gastrointestinal cancers by immunohistochemistry or by molecular MSI are used as biomarkers in the current clinical setting, while mutations in DNA polymerase genes (POLE, POLD1) could become predictive biomarkers in endometrial and colon cancer in the future [33, 34].

Similarly, the loss of DNA methylation of certain genes including the cancer testis antigens (CTAs) and their consequent upregulation in tumor cells (e. g. *MAGE-A1, MAGE-A3, NY-ESO-1, SSX-2, SSX-4*) might indicate a better response to immune checkpoint blockade, as these antigens may be recognized as non-self structures [35–37].

On another note, hyperprogression, which is found rarely but repeatedly during treatment with immune checkpoint inhibitors might be correlated with *MDM2/MDM4* amplification, *EGFR *alterations and/or *DNMT3A* mutations, which might lead to early recognition, if properly validated.

## Radiologic investigations in immune checkpoint inhibitor treatment

Radiologically, tumor size is the easiest parameter for the determination on an effect of any anticancer treatment. Although this also applies to treatment with immune checkpoint inhibitors, there are particular treatment responses, which are not observed in patients receiving other kind of treatments. As immunotherapies do not target directly the tumor cells but the immune system, the time to a measurable tumor response can be variable. Therefore, in some patients the tumors may remain stable in size or even grow slowly over some weeks or even months before they show a decrease in size. In other patients, the infiltration of tumors by inflammatory cells leads to a temporary increase in tumor size. This so-called pseudoprogession is observed in 10–15% of patients with melanomas and in less than 2% of patients with lung cancer and must not be confused with treatment failure. In analogy, even new lesions might become visible during immune checkpoint blockade, which could be the result of an ensuing visibility of previously undetected metastases due to lymphocyte infiltration. To discriminate progressive disease from pseudoprogression, short-term follow-up examinations not earlier than 4 weeks after the examination in which a progression of disease was observed are advised. Thus, the course of disease under treatment with immune checkpoint inhibitors should be monitored by comparing serial and repeated measurement of target lesions before treatment is abandoned, always keeping in mind the biological principles of treatment versus tumor control. This, however, only applies to patients with no clinical deterioration.

For study purposes, a number of response criteria have been developed, the first one being the immune-related response criteria (irRC) published in 2009 [38]. Very recently, new immune response criteria in solid tumors (iRECIST) criteria have been published, which will be used in future prospective trials in addition to conventional response criteria [39, 40].

## Adverse events and side effects management

Immunotherapy by checkpoint inhibition can cause immune-related adverse events (irAEs) in a considerable number of patients due to the induction of overstimulation of immune reactivity or to the generation of outright autoimmune phenomena [41]. With CTLA-4-inhibition, such side effects are observed in up to 7 patients out of 10, while with PD-(L)1 inhibitor treatment, these occur in only 2–3 out of 10 [42]. As immunotherapy in cancer is assumed to activate the tumor-directed T‑cell response by T‑cells infiltrating the primary tumor and its metastases, this therapeutic intervention can also cause irAEs in all types of tissues. These irAEs may include the induction of diarrhea or the emergence of ulcerative colitis or Crohn’s disease, Hashimoto’s thyroiditis, autoimmune hepatitis, uveitis and hypophysitis which can be life-threatening complications if not recognized and treated appropriately [41, 43, 44].

With rash and pruritus often occurring as the first side effect of anti CTLA-4 treatment, liver toxicity, diarrhea, colitis and hypophysitis tend to appear later. In PD-(L)1 inhibition, most common irAEs are cutaneous and gastrointestinal, less common endocrine, hepatic, pulmonary and renal. Combination of checkpoint inhibitors and duration of therapy cause more severe adverse events typically associated with those encountered during CTLA-4 immune checkpoint inhibition.

In patients receiving immune checkpoint inhibition treatment, every symptom has to be suspected to represent a sign of a possible irAE, and patients should be informed that they should contact the hospital once a possible side effect occurs. Similarly, the patients’ general practitioners should have basic information about irAEs. Early diagnosis and onset of treatment can prevent the development from grades 1–2 to grades 4–5 toxicities. At the hospital, an interdisciplinary team should be ready to assess and manage side effects of immunotherapy according to published management algorithms. While grade 1, irAEs should be managed symptomatically under continued PD-(L)1 inhibition, grades 2 and 3 toxicities necessitate delay of treatment plus the addition of 1–2 mg prednisone/kg body weight with tapering to a dosage of below 10 mg. Grade 4 events and recurrent grade 3 events should cause permanent discontinuation of PD-(L)1 inhibition [45, 46].

## Overcoming treatment resistance

When following patients under immune checkpoint inhibitor treatment, it became clear that primary and adaptive resistance to immunotherapy might occur which limits the efficacy of treatment. This calls for concepts to maximize the clinical benefit of immune checkpoint inhibitor treatment by the combination with either other immunotherapy, with chemotherapy, with radiotherapy or with targeted therapies using tyrosine kinase inhibitors. For all of these therapeutic approaches, an abundance of clinical trials in a similar abundance of clinical scenarios and diseases are under way. Aims are to not only increase the activation and function of immune cell-associated tumor cell destruction, but also to widen the current concept to other immune cells including T regulatory cells, myeloid-derived suppressor cells and neutrophils, and, finally, make tumors more immunogenic and induce their infiltration by immunocompetent cells. Overcoming resistance mechanisms will be the key for the obtainment of an enhanced efficacy of immunotherapy in cancer [47].

Primary and intrinsic mechanisms of resistance or mechanisms of adaptation as well as secondary or acquired resistance are the limiting factors for immune checkpoint inhibitor treatment. Thus, gene editing with amplification of PD-L1 in malignant cells appears to occur early and makes malignant cells prone to respond to PD-(L)1 inhibitors, whereas the loss of PD-L1 seems to be the cause of resistance to treatment occurring in some patients. Thus, future strategies should not only address the malignant cells themselves but also their microenvironment and the function of immunocompetent cells [48, 49]. These strategies are given here:

### Combinations of immune checkpoint inhibitor treatment with other immunotherapies

The idea of addressing the immune response and the tumor microenvironment has been studied in the Checkmate 012 study in first line treatment of stage IV NSCLC, as CTLA-4 inhibition by ipilimumab is directed at the microenvironment, which should increase the efficacy of nivolumab seen in this disease [51]. The proof of this concept was generated by the fact that in a subgroup of patients, a partial response was observed in 39% with PFS at 24 weeks seen in 63% of patients; however, an abundance of other immunomodulating compounds, such as vaccines in combination with immune checkpoint inhibitors and antagonistic antibodies directed against proteins, such as LAG-3 or activating antibodies against such peptides as OX-40 are in current clinical testing [52].

### Combinations of immune checkpoint inhibitors with chemotherapy

Checkpoint inhibition plus chemotherapy may show additional benefits as cytotoxic drugs have more effects than only causing death of malignant cells thereby setting free cancer-associated antigens but also by interfering with the function of a series of immunocompetent cells. Thus and as examples, anthracyclines augment dendritic cell activation, cyclophosphamide promotes anti-tumor CD4+ cells and cell recognition and lysis by CD8+ cells, and cisplatin abrogates the activity of T‑regulatory cells and myeloid-derived suppressor cells [53–55].

### Combination of immune checkpoint inhibitors with tyrosine kinase inhibitors

In the combination of targeted therapies with immune checkpoint inhibition, early studies using ipilimumab and vemurafenib have shown that care has to be taken regarding potential severe toxicities, particularly hepatotoxicity [56]. This seems to depend on the drugs of choice and the sequence of their application as, for an impressive example, the combination of nivolumab with VEGF inhibitors or TKIs may decrease the number and function of T‑regulatory cells. The disruption of angiogenesis under the mentioned treatment plus immune checkpoint inhibition has shown to exert important efficacy in the treatment of advanced RCC.

### Combination of immune checkpoint inhibitors with radiotherapy

An abundance of clinical trials are under way do determine the effect of a combination of checkpoint inhibitors with radiotherapy which have been primarily observed by the induction of an abscopal effect resulting from antigen exposure originating from destroyed cancer cells and the induction of a type I IFN response [57, 58].

## Limitations to access

The actual status and future perspectives of immunotherapy in cancer depend on the access of patients to these innovative treatments. Even in the most affluent member states of the EU, there are considerable differences in this field. With currently 28 EU member states and their 28 various healthcare and reimbursement systems and 28 various interpretations of the EU Cross-border Directive make access to checkpoint inhibitor treatment complex and diverse. New pricing models could be a future way for reimbursement in order to simplify the access to this important class of drugs [59–61]. The involvement of patients and patient advocacy groups must become the norm in HTA assessment which should serve the patients to get access to innovative drugs, as outlined in the magnitude of clinical benefit scale generated by the European Society for Medical Oncology [62].
